# Numerical Study of Multivortex Regulation in Curved Microchannels with Ultra-Low-Aspect-Ratio

**DOI:** 10.3390/mi12010081

**Published:** 2021-01-14

**Authors:** Shaofei Shen, Mengqi Gao, Fangjuan Zhang, Yanbing Niu

**Affiliations:** College of Life Science, Shanxi Agricultural University, Taigu 030801, China; tianchang984@nwsuaf.edu.cn (M.G.); panglong2012@nwsuaf.edu.cn (F.Z.)

**Keywords:** curved microchannel, inertial microfluidics, Dean flow, secondary flow, multi-vortex regulation

## Abstract

The field of inertial microfluidics has been significantly advanced in terms of application to fluid manipulation for biological analysis, materials synthesis, and chemical process control. Because of their superior benefits such as high-throughput, simplicity, and accurate manipulation, inertial microfluidics designs incorporating channel geometries generating Dean vortexes and helical vortexes have been studied extensively. However, existing technologies have not been studied by designing low-aspect-ratio microchannels to produce multi-vortexes. In this study, an inertial microfluidic device was developed, allowing the generation and regulation of the Dean vortex and helical vortex through the introduction of micro-obstacles in a semicircular microchannel with ultra-low aspect ratio. Multi-vortex formations in the vertical and horizontal planes of four dimension-confined curved channels were analyzed at different flow rates. Moreover, the regulation mechanisms of the multi-vortex were studied systematically by altering the micro-obstacle length and channel height. Through numerical simulation, the regulation of dimensional confinement in the microchannel is verified to induce the Dean vortex and helical vortex with different magnitudes and distributions. The results provide insights into the geometry-induced secondary flow mechanism, which can inspire simple and easily built planar 2D microchannel systems with low-aspect-ratio design with application in fluid manipulations for chemical engineering and bioengineering.

## 1. Introduction

Microfluidics is generally known as an advanced technology that deals with fluids and microparticles/cells under precise manipulation [1,2]. Microfluidic systems are also considered as platforms for solving problems in biomedicine, materials synthesis, and biochemical reactions [3,4,5]. Many techniques have been proposed and developed for fluid control in microfluidic systems [6,7]. Depending on the source of the manipulative forces, these compelling techniques have advantages over traditional macro-scale platforms and can be divided into active and passive types. Active techniques are dependent on external energy sources, while passive techniques rely entirely on internal hydrodynamics or channel geometry [8,9]. An active microfluidic technique commonly allows for a more accurate manipulation of target samples and real-time tunability. However, the flow rate is always constrained due to the two dominant competitions of external force field and hydrodynamic drag forces. Additionally, it is difficult to produce and integrate other microfluidic components [9,10,11]. In contrast, a passive technology can overcome these drawbacks and is more easily fabricated, operated, and maintained, and can operate at a higher flow rate [11,12]. Inertial microfluidics making use of geometry-induced vortexes have recently attracted significant attention because of their continuous and efficient processing, simple structure, low cost, increased fluidic controllability, and high throughput [13,14,15].

Various channel designs that manipulate vortex actions to increase the interface area of fluid streams, depending on non-conventional applications of fluid inertia in microfluidic platforms, have been demonstrated [13,15]. The vortex usually appears in (i) straight channels with expansion–contraction arrays or disturbance obstacles [16,17,18,19], and (ii) curved channels with spiral or serpentine geometry [20,21,22,23,24]. Due to the fluid momentum mismatch between the near wall and the central area in these platforms, Dean vortex or Dean-like secondary vortex flows can be generated in the main channel section by a radial pressure gradient [13]. Besides the formation of a secondary counter-rotating vortex flow, channels with disturbance obstacles can also generate a horizontal micro-vortex under a high flow rate because of the detachment of the boundary layer, and have been employed in fluid and particle manipulations [25,26,27,28,29,30]. However, such unique channel designs often face the challenge of high microchannel production and cost increase because of the standard soft lithography and stereolithography technology [18]. Currently, compared with other approaches to producing microfluidic devices (e.g., micromachining, micromilling, hot embossing, and injection molding), the standard soft lithography and stereolithography technology is the most promising method for the routine creation of microfluidic structures [31,32,33,34,35,36]. Therefore, a low-aspect-ratio (AR) channel should be developed instead of a high-AR channel. Our group recently established a unique approach using ultra-low-AR microchannels for regulating the Dean vortex to achieve highly efficient fluid mixing and cell manipulation [37,38,39]. The systematic investigation and demonstration of multi-vortexes in vertical and horizontal planes have been less advanced. A simple and effective way to induce and accelerate multi-vortexes in ultra-low-AR curved microchannels with a simple configuration and easy production has remained largely out of reach. In this study we expand our work to systematically explore the generation and regulation mechanism of a multi-vortex by modifying the number of obstacles, the operational flow rate, the micro-obstacle length, and the channel height in the ultra-low-AR curved microchannels.

Using a unique integration of a sequence of ordered micro-obstacles and a semicircular channel, an ultra-low-AR (1:9) microchannel platform with a single layer is developed. Multi-vortex formation in the horizontal and vertical planes of the curved microfluidic is primarily highlighted to increase the processing performance and throughput. We systematically study the mechanism of the multi-vortex and the underlying physics in the semicircular channel with different distributions of ordered micro-obstacles. Outstanding acceleration of the multi-vortex can be produced by increasing the operational flow rate and the length of sequenced micro-obstacles in the semicircular channels. The Dean vortex can be regulated to become larger by decreasing the channel height. Through numerical simulation, we demonstrate that the purposeful regulation of dimensional confinement in the curved microchannel can induce a Dean vortex and a helical vortex with different magnitudes.

## 2. Experimental Methods

In order to assess fluid motion in the microfluidic control system, ESI-CFD software was used to simulate computational fluid dynamics (CFD) (V2016.0, ESI CFD, Inc., Huntsville, AL, USA). Steady-state incompressible flow was applied. In this experiment, various flow rates (0.01, 0.02, 0.05, 0.1, 0.2, 0.5, 1, 2, 5, 10, 15, and 20 mL min^−1^) were set at the inlet boundary condition, and the outlet was set as a fixed pressure boundary condition with no viscous stress condition to obtain the steady flow field. There was a no-slip boundary condition on the groove wall. For the exploration of fluid phenomena in the microchannels, FLOW modules in CFD-ACE+ were employed. Multiblock structured meshes around two million cells were employed. Structured and unstructured meshes were applied in the numerical analysis. In the setting of curve and surface meshing, triangle global was selected as the mesh type. The max cell size and min cell size were 50 and 2, respectively. The curve mesh transition factor was 1.1. The physical properties of water (density *p* = 1000 kg m^−3^ and dynamic viscosity *µ* = 10^−3^ kg m^−1^ s^−1^) were used for the fluids taking part in the computational fluid dynamics simulation. A diffusion coefficient *D* = 10^−10^ m^2^ s^−1^ was used for the fluids in the simulations.

## 3. Results and Discussion

### 3.1. Theory and Design Principle

It is well-known that the unique helical vortex is generally induced by the micro-obstacle arising at the corner of the expansion chamber in the expansion–contraction channel [19]. The helical vortex is a rotational flow that exists in the horizontal plane of the channel. The helical vortex is distinct from the Dean vortex in a curved channel and asymmetrically structured channel [14,18]. The formation of helical vortexes depends on several factors, including the ratio of the contraction area to the expansion area, shape and size of the micro-obstacle, the flow velocity, and the fluid inertia [13,16]. It is noteworthy that the fluid inertia is mainly dependent on *Re*.

The Dean vortex induced in the vertical planes of a curving channel by a pressure gradient is a well-known inertial effect caused by the mismatch of fluid momentum in the center of the channel and the area near the wall. Fluid elements near the center line of the channel flow faster and have more momentum than those near the wall surface of the channel. They are inclined to flow outward along the curve and form a pressure gradient along the radial direction in the channel. Since the channel is closed, to conserve mass, relatively stagnant fluid near the top and bottom walls recirculates inward because of this centrifugal pressure gradient, finally forming two counter-rotating symmetric streams [14]. The magnitude and qualitative aspects of the Dean vortex features a dimensionless parameter, the Dean number; *De* = *Re*(*D_h_*/2*R*)^0.5^, where R denotes the channel curvature’s radius. The Dean vortex’s strength is dependent on the geometrical ratio *D_h_*/2R, and the underlying downstream flow (*Re*)’s magnitude, where *R* denotes the curvature radius, *D_h_* denotes the channel’s hydraulic diameter, and *Re* denotes the channel Reynolds number [37].

**Figure 1:**
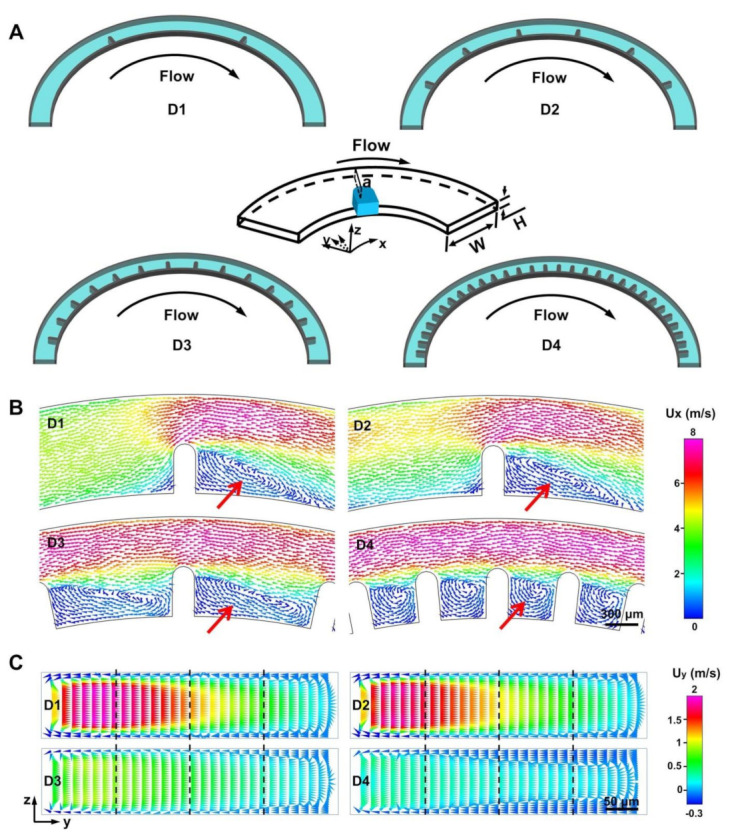
Multi-vortex regulation based on dimensional confinement. (**A**) Configuration of the four dimension-confined curved microfluidic channels (D1, D2, D3, D4). The D-channels are equipped with different amounts of ordered micro-bars. In the D-channels, the narrow regions are all 450 μm wide, and the wide regions are all 900 μm wide. The channel heights are 100 μm. (**B**) Representative helical vortex induced by micro-bars along the D-channels (D1, D2, D3, D4) under flow rates of 15 mL min^−1^. The red arrows represent different vortex distributions. (**C**) Simulated demonstration of the Dean flow acceleration in the y-axis along channel cross sections of the narrow regions of the D-channels (D1, D2, D3, D4). The flow rate is 15 mL min^–1^. To evaluate the local flow field quantitatively, the section is divided into four parts.

Based on the above analysis, it can be said that the qualitative characteristics and magnitude of two vortexes are significantly influenced by channel construction apart from the *Re*, serving as key factors to design channel structure for preferable fluid and particle applications. More specifically, the parameters of microchannel construction including the ratio of *D_h_* to *R*, H/W (H represents the channel’s height and W represents the channel’s width), and the various heights of the inner and outer wall significantly affect the multi-vortex’s distribution and strength [12,13]. However, the strategy for designing geometries for producing multi-vortexes basically focuses on a high-AR expansion–contraction channel, because the weakening of the Dean and helical vortex effect as the height decreases [38]. To better explore the inertia mechanism of the multi-vortex in a low-AR microchannel, a semicircular microchannel with an ultra-low AR (H/W = 100 μm/900 μm) and 6000 μm radius of curvature was developed. In addition to this, the arranged micro-obstacles in the channel (namely a dimension-confined curved channel, D-channel) were set as a dependent regulator to adjust the distribution and magnitude of the multi-vortex. The arranged micro-obstacles are added only to the inner wall of channel which can take better advantage of the Dean vortex generated in the normal semicircle channel for fluid and particle manipulations [37,38]. Four D-channels (D1, D2, D3, and D4) with the same channel length are designed with different amounts of ordered micro-obstacles (2, 6, 14, 30) respectively (Figure 1A). To identify the channel position, we defined a coordinate system (x, y, z), which is the same as that in the previous study on the curved microfluidic system [37,38]. In the designed system, the z-axis points to the roof along the channel depth. The y-axis always points to the channel wall perpendicular to the x-axis, while the x-axis refers to the main flow direction from the inlet to the outlet (Figure 1A). Various distributions of the helical vortex in the horizontal plane (Figure 1B) and the Dean vortex in the vertical plane (Figure 1C) could be produced in the four D-channels (micro-bar length = 450 μm) by numerical simulation. Further investigations of the multi-vortex features in the designed ultra-low-AR microchannels are illustrated in the following.

### 3.2. Helical Vortex and Dean Vortex Formations

**Figure 2:**
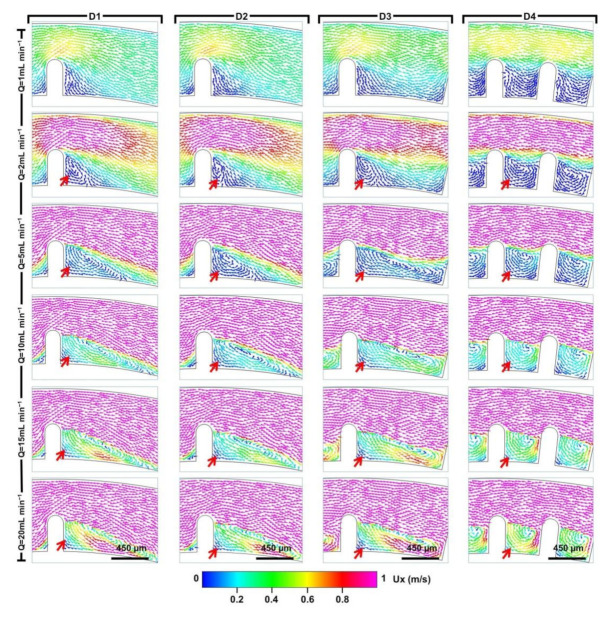
Simulated fluid velocity field of helical vortex induced by micro-bars along the D-channels (D1, D2, D3, D4) under various flow conditions (1 to 20 mL min^−1^). The red arrows represent different vortex distributions. Ux represents the fluid velocity field in the horizontal plane and refers to the main flow direction from the inlet to the outlet.

Numerical simulation was further applied to qualitatively study the fluid motion in the four designed D-channels (micro-obstacle length = 450 μm). As shown in Figure 2, at different flow rates from 0 to 10 mL min^−1^, pronounced helical vortex variations in the horizontal plane were observed with variations of the fluid velocity in the vortex region.

When the flow rate was 1 mL min^−1^, no helical vortex appeared in the four D-channels. As the flow rate rose to 2 mL min^−1^, a small helical vortex zone appeared behind the micro-obstacle and formed different regional distributions. The regions of the helical vortexes in D3 and D4 were obviously larger than those in D1 and D2 due to the configuration differences of the micro-obstacles. At a higher flow rate of 5 mL min^−1^, a significant helical vortex zone was observed. The range of the vortex regions became wider in all D-channels except for D4. This was probably because a narrow space between the micro-obstacles in D4 resulted in the maximum regional distribution under this flow rate. When the flow rates were ≥10 mL min^−1^, vortex distributions of the other D-channels were also extended to the maximum extent. However, the magnitude of the fluid velocity in the vortex region of the four D-channels increased gradually with increased operational flow rate. In summary, it was demonstrated that the magnitude and distribution features of the helical vortex could be generated and regulated by changing the space between the micro-obstacles and the operational flow rate.

**Figure 3:**
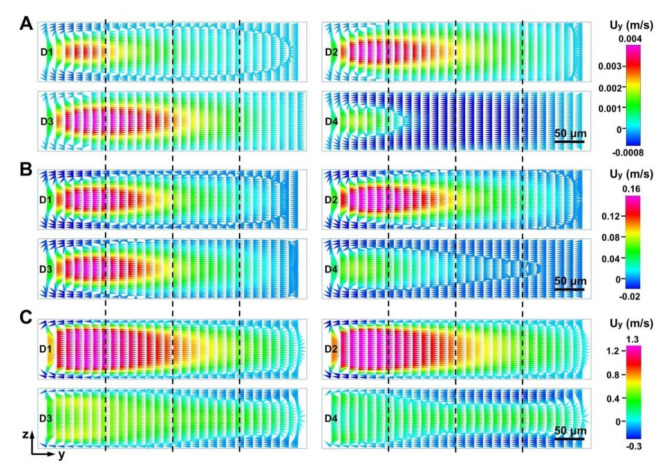
Dean flow acceleration in the y-axis along channel cross sections of the narrow regions (narrowing ratio (NR) = 450/900) of the D-channels (D1, D2, D3, D4) under different flow rates of 0.1 mL min^−1^ (**A**), 1 mL min^−1^ (**B**), 10 mL min^−1^ (**C**), respectively.

**Figure 4:**
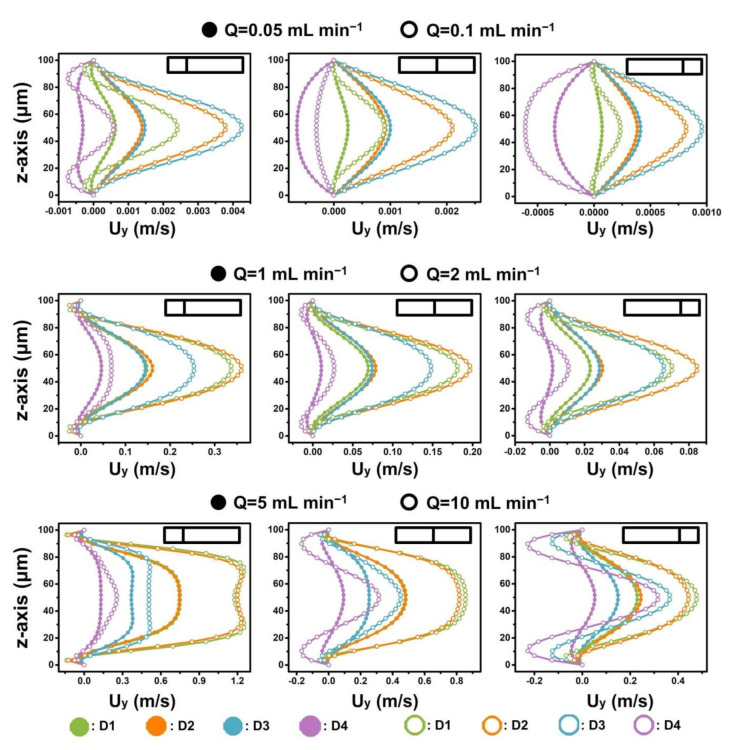
Quantitative analysis of fluid velocity along channel cross sections in narrow regions of different D-channels under different flow rates (0.05 to 10 mL min^−1^). The partial results correspond to the dotted lines in Figure 3.

When fluid flowed into the narrow regions of four D-channels (micro-obstacle length = 450 μm), the magnitude and distribution of the Dean vortex in the narrow regions of the four D-channels were different and complex (Figure 3 and Figure 4, and Supporting Information Figure S1). To better distinguish flow dynamics in the D-channels, the cross section was quartered to calculate the simulated velocity field along the border (Figure 1C and Figure 3). The fluid velocity in the y-axis (U_y_) and the x-axis (U_x_) became higher with increasing operational flow rate (Figure 4 and Supporting Information Figures S1–S3). The U_x_ values in the D-channels were almost similar at the same given operational flow rate range (0.01–5 mL min^−1^). However, the fluid velocities in the y-axis (U_y_) of the D-channels varied. D4 had the maximum micro-obstacle distributions yet had the lowest U_y_. When the flow rate increased to a higher range (10–20 mL min^−1^), the U_x_ in the left border of D1 and D2 became slightly higher than those in D3 and D4, yet U_x_ in the middle and right borders of D1 and D2 were slightly lower than those in D3 and D4. It is noteworthy that the U_y_ values along borders in D1 and D2 were many times higher than those in D3 and D4 (Figure 4 and Supporting Information Figures S1–S3). D3 had the highest U_y_ when the flow rates were 0.01–0.5 mL min^−1^. D2 had the highest U_y_ when the flow rates increased to 1–5 mL min^−1^, and D1 had the highest U_y_ when the flow rates increased to 10–20 mL min^−1^. The calculated results revealed that the intensity of the Dean vortex could be regulated by controlling the number of micro-obstacles and the operational flow rate. As the flow rate increased, reducing the number of micro-obstacles in the D-channels significantly promoted the Dean vortex in the narrow cross section. However, the resulting reduction in the number of Dean vortexes negatively affected fluid manipulations. Accordingly, it is vital to yield the optimal number of micro-obstacles and flow rate when studying fluid applications [16,17]. These findings verify the value in using sequenced micro-obstacles in curved channels to modify the Dean vortex achieved and lay the theoretical basis for fluid mixing and particle manipulation [37,38]. Further studies on the responses of multi-vortexes to micro-bar length and AR alteration based on the device are presented in subsequent sections.

### 3.3. Multivortex Regulation by Micro-Bar Length and AR Alteration

**Figure 5:**
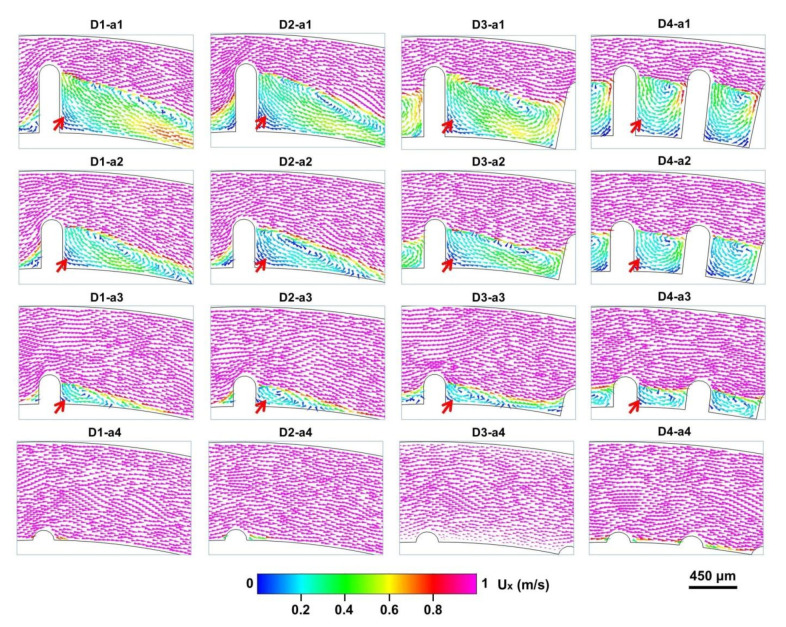
Simulated fluid velocity field of the helical vortex induced by micro-obstacles by arrow distribution in the x-axis of the D-channels (D1, D2, D3, D4) at a flow rate of 15 mL min^−1^. a1, a2, a3, and a4 represent 270, 450, 630, and 810 μm width of the narrow regions in Figure 1A respectively. The red arrows represent different vortex formations in the horizontal.

For a deep exploration of multi-vortex regulation, we designed four microbar lengths (90, 270, 450, and 630 μm) in the D-channels. The structure system was classified according to various channel narrowing ratios (NRs) (channel width of the narrow region divided by the channel width of the wide region), including D-channels (NR = 810/900 (D1-a4, D2-a4, D3-a4, D4-a4), 630/900 (D1-a3, D2-a3, D3-a3, D4-a3), 450/900 (D1-a2, D2-a2, D3-a2, D4-a2), 270/900 (D1-a1, D2-a1, D3-a1, D4-a1)). A comparative experiment of the helical vortex distribution induced by micro-obstacles was conducted in the x-axis of the D-channels at 15 mL min^−1^ (Figure 5). The results indicate that a pronounced helical vortex in the micro-obstacle corners could be triggered by introducing micro-obstacles of different lengths (270, 450, and 630 μm) in the D-channels (D1, D2, D3, D4). It was observed that the longer the length of the micro-obstacle, the stronger the helical vortex generated. It is noteworthy that the helical vortex could be induced by shorter micro-obstacles (length = 90 μm) in the horizontal of D-channels (NR = 810/900 (D1-a4, D2-a4, D3-a4, D4-a4)) under the high operational flow rate.

**Figure 6:**
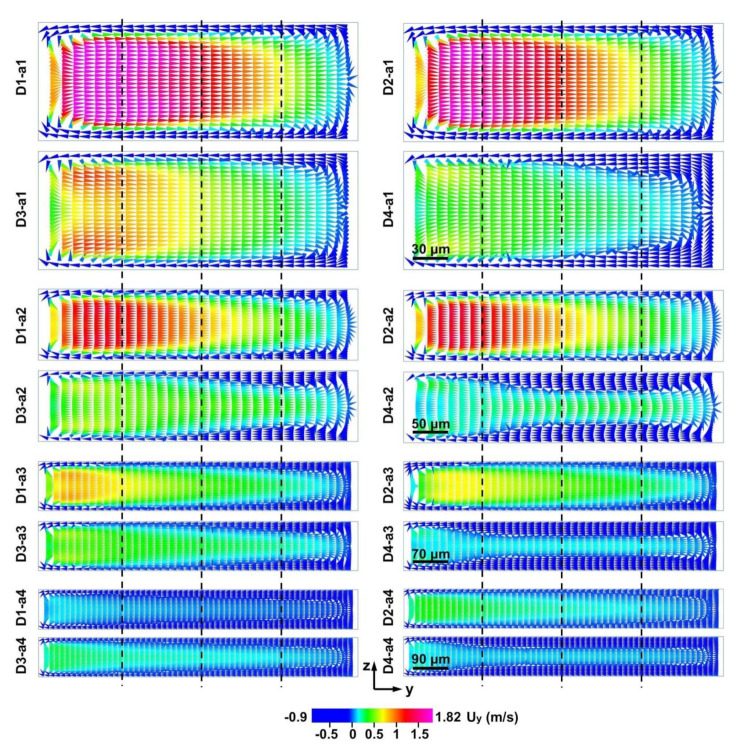
Simulated Dean flows in the y-axis along channel cross sections of the narrow regions (NR = 270/900, 450/900, 630/900, and 810/900) of the D-channels (D1, D2, D3, D4) at a flow rate of 15 mL min^−1^. a1, a2, a3, and a4 represent 270, 450, 630, and 810 μm widths of the narrow regions in Figure 1A respectively. To evaluate the local flow field quantitatively, the section is divided into four parts.

**Figure 7:**
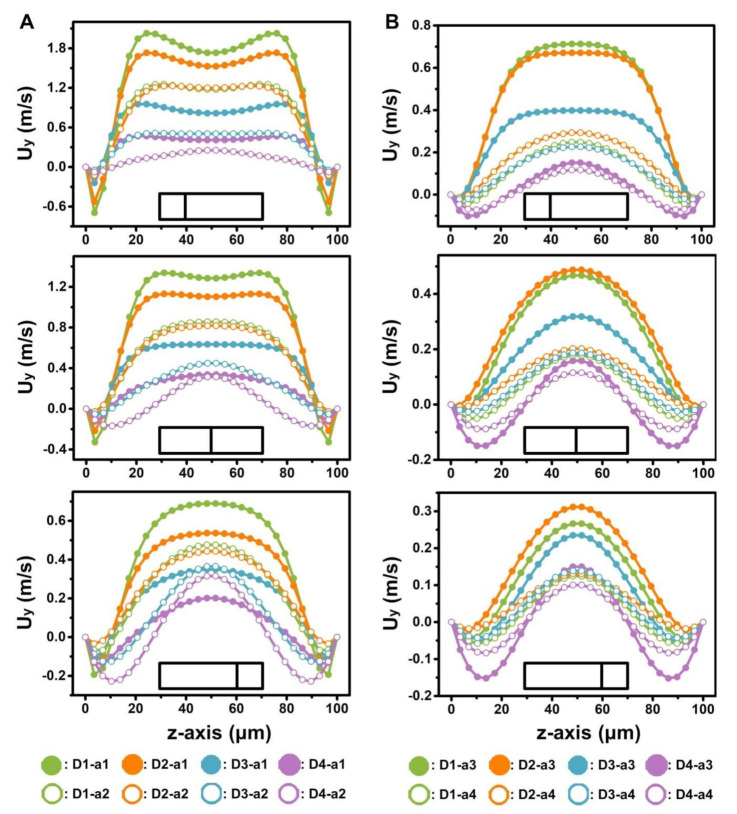
(**A**,**B**) Quantitative analysis of fluid velocity along channel cross sections of D-channels (D1, D2, D3, D4) with different widths of the narrow regions (a1 (**A**), a2 (**A**), a3 (**B**), a4 (**B**)) at a flow rate of 15 mL min^−1^. The results correspond to the solid lines in Figure 6. a1, a2, a3, and a4 represent 270, 450, 630, and 810 μm widths of the narrow regions in Figure 1A respectively.

Moreover, U_x_ showed minimal difference among the narrow regions with the same width (a1, a2, a3, a4) in the different D-channels when fluid entered the narrower regions under identical initial flow rate (15 mL min^−1^) (Supporting Information Figures S4 and S5). However, the Dean vortexes in the narrow regions of the D-channels were notably different (Figure 6). The U_y_ in the narrow regions increased with decreased NR in the D-channels (Figure 6 and Figure 7). This result demonstrates that the micro-obstacle installation brought about a noteworthy acceleration of the Dean vortex.

More obvious curvature radius variation from the wide (6000 μm) to narrow (6090 μm representing a-4; 6270 μm representing a-3; 6450 μm representing a-2; 6630 μm representing a-1) regions are likely to accelerate the Dean vortex in the narrow region significantly [37]. Furthermore, the intensity of the Dean vortex could be adjusted by controlling the number of micro-obstacles when the micro-bar lengths were 270, 450, and 630 μm. Shorter micro-obstacles (length = 90 μm) in the D-channels (NR = 810/900 (D1-a4, D2-a4, D3-a4, D4-a4)) could not induce an organized Dean vortex distribution. These results demonstrate the intensity of the helical vortex and that the Dean vortex can be adjusted by regulating the confinement degree. From the above results, the NR (namely, micro-bar length) can serve as an effective factor for adjusting the magnitude of the multi-vortex in curved channels.

**Figure 8:**
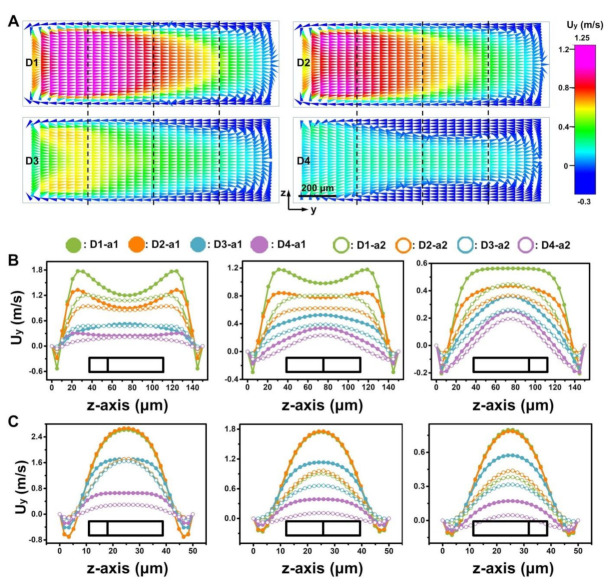
Fluid motion along channel cross sections in narrow regions of the D-channels at a flow rate of 15 mL min^−1^. (**A**) Simulated Dean vortex in the y-axis of a narrow region (NR = 450/900) in different D-channels (D1, D2, D3, D4) with a 150 μm height. To evaluate the local flow field quantitatively, the section is divided into four parts. (**B**,**C**) Quantitative study of the y-axis fluid velocity in each narrow region (NR = 270/900, 450/900, 630/900, 810/900) of different D-channels (D1, D2, D3, D4) with 150 μm (**B**) and 50 μm (**C**) height. a1, a2, a3, and a4 represent 270, 450, 630, and 810 μm widths of the narrow regions in Figure 1A respectively.

Apart from the length of micro-obstacles, AR alteration is suggested to play an important role in multi-vortex regulation. Therefore, comparisons between the Dean vortex in the D-channels with different heights (50, 150 μm) in the narrow regions were studied. Despite having similar U_x_ values in the narrow regions with the same width (a1, a2, a3, a4) for the D-channels with different heights (Supporting Information Figure S6), much stronger Dean vortexes were induced with a height decrease in these D-channels (NR = 270/900, 450/900, 630/900). Only D-channels D1-a4, D2-a4, D3-a4, and D4-a4 accelerated the Dean vortex by increasing the height (Figure 8 and Supporting Information Figure S7), which was similar to normal curved channels (namely, no dimension-confined curved channel).

This mainly results from the fact that minor modifications of the radius of curvature of the D-channel (NR = 810/900) from wide to narrow regions cannot give rise to the large changes in the fluid path. Generally, the increment of channel height leads to an increase of the *D_h_*, causing stronger Dean vortexes in normal curved channels at a given flow rate [38,40]. The consequences of Dean vortex acceleration are favorable for fluid manipulation applications, which is a critical reason why normal curved channels cannot be designed using an ultra-low AR [41,42].

In contrast, the accelerated Dean vortex in D-channels was caused by channel geometry changes (e.g., the height and curvature of the channel). While this was a highly technical quantitative analysis, further in depth investigation is required [13,15]. With regards to the comparison between various D-channels, the Dean vortex diminishes with decreasing NR because the radius of curvature from wide to narrow decreases.

## 4. Conclusions

This study focused on the deterministic regulation of the helical vortex and the Dean vortex in a dimension-confined semicircular channel. We systematically studied the generation and regulation mechanism of multi-vortexes by the number of obstacles, the operational flow rate, the micro-obstacle length, and the channel height in the designed ultra-low-AR curved microchannels. Through numerical simulation, the regulation of dimensional confinement in the microchannel was demonstrated to induce Dean and helical vortexes with different magnitudes and distributions. The channel narrowing ratios can offer a simple method to develop microfluidic systems for precise manipulation of the Dean vortex. Benefiting from the precise generation and regulation of multi-vortexes the results can provide insights into the geometry-induced secondary flow mechanism, inspiring simple and easily fabricated planar 2D microchannel systems with low-AR design. This system enables easy fabrication and use, is high-throughput, and has considerable flexibility in the operation of fluid manipulation for chemical engineering and bioengineering. It is anticipated that the inertial strategy could enhance the conceptual understanding of helical vortex and Dean vortex manipulation and offer insight into ultra-low-AR microchannel design.

## Data Availability

Data sharing is not applicable to this article.

## Supplementary Materials

The following are available online at https://www.mdpi.com/2072-666X/12/1/81/s1.
