# Supplementary material for: Numerical Study of Multivortex Regulation in Curved Microchannels with Ultra-Low-Aspect-Ratio
Source: Micromachines (Basel). 2021 Jan 14;12(1):81. doi: 10.3390/mi12010081 (PMC7830345; doi:10.3390/mi12010081)
Supplement: Supplementary file 1 [file micromachines-12-00081-s001.pdf]

micromachines

Supplementary Information

Numerical Study of Multivortex Regulation in Ultra-low Aspect Ratio Curved Microchannels

Shaofei Shen *, Mengqi Gao, Fangjuan Zhang and Yanbing Niu *

College of Life Science, Shanxi Agricultural University, Taigu 030801, China; tianchang984@nwsuaf.edu.cn (M.G.); panglong2012@nwsuaf.edu.cn (F.Z.)
* Correspondence: shenshaofei@nwafu.edu.cn (S.S.), zhaol@nwafu.edu.cn (Y.N.); Tel./Fax: +86-354-6287205 (S.S. & Y.N.)

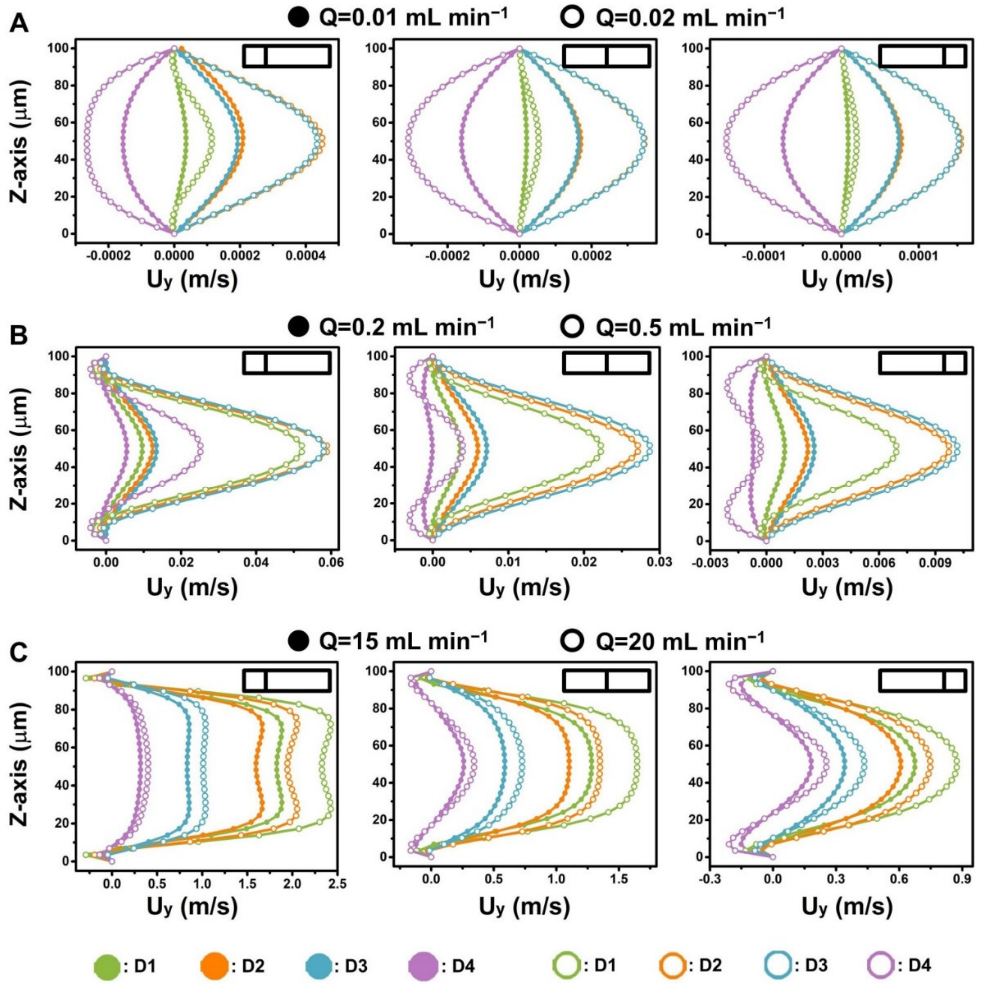

Figure S1. Quantitative analysis of fluid velocity field along channel cross-sections of narrow regions of the D-channels under different flow rates (0.01 to 20 mL min^{-1}). The partial results correspond to the dotted lines in Figure.1C.

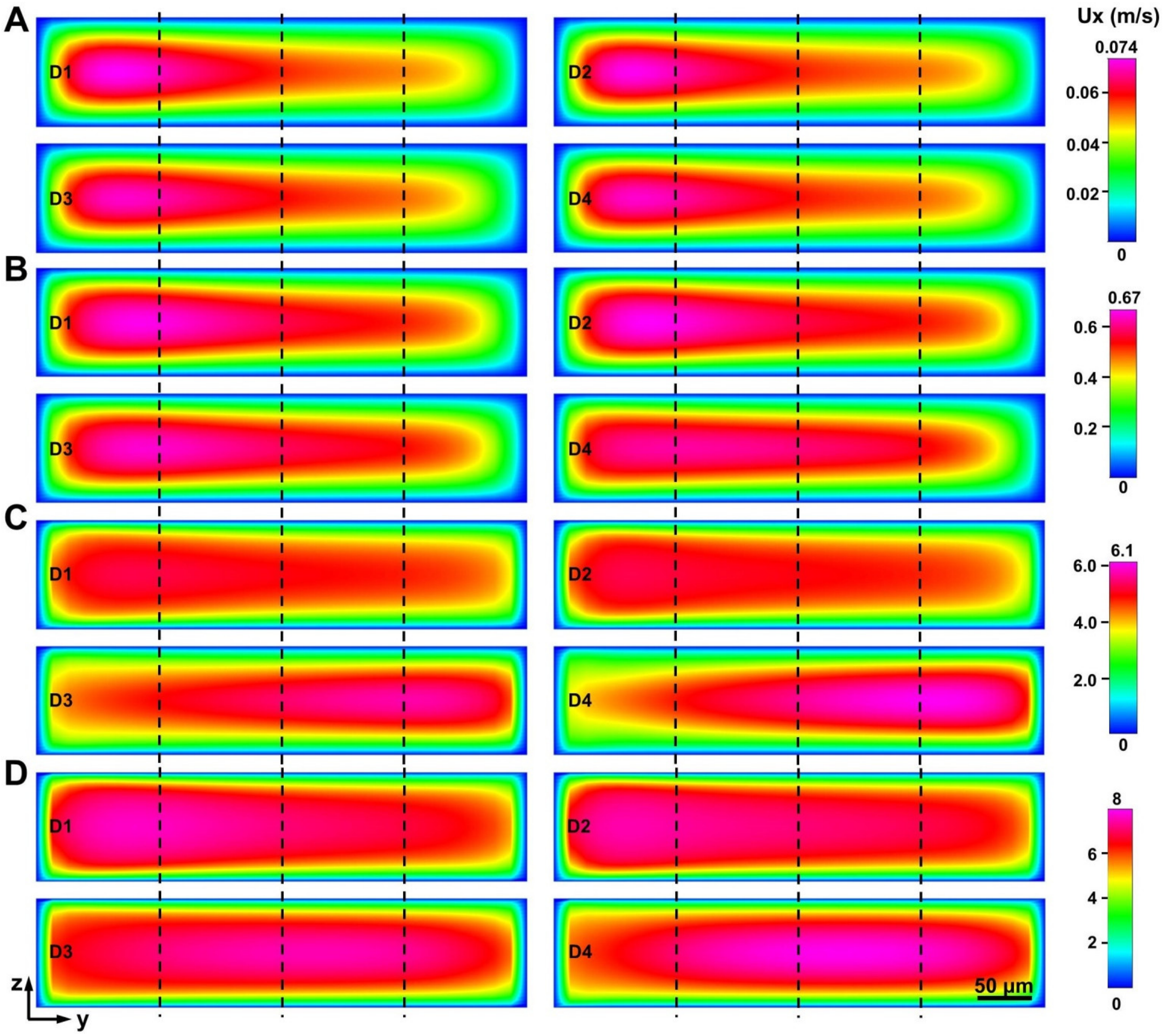

Figure S2. Simulated fluid velocity field in x-axis along channel cross-sections of narrow regions of the D-channels under different flow rates of 0.1 mL min^{-1} (**A**), 1 mL min^{-1} (**B**), 10 mL·min^{-1} (**C**), 15 mL min^{-1} (**D**), respectively. The cross-sections were quartered for quantitative evaluation of local flow field.

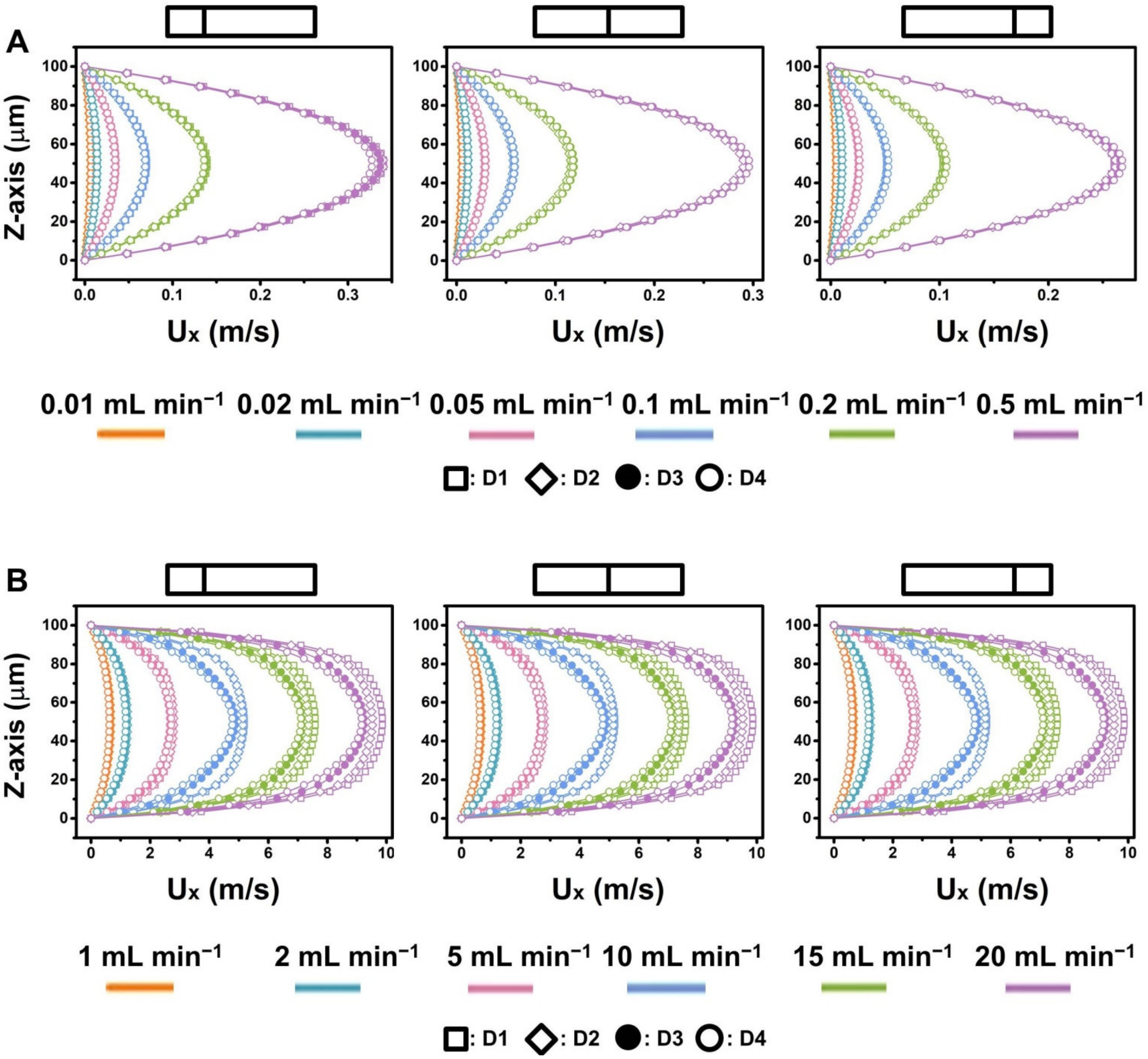

Figure S3. Quantitative analysis of fluid velocity in x-axis along channel cross-sections of narrow regions of the D-channels different flow rates (0.01 to 20 mL min^{-1}). The partial results correspond to the dotted lines in Figure S2.

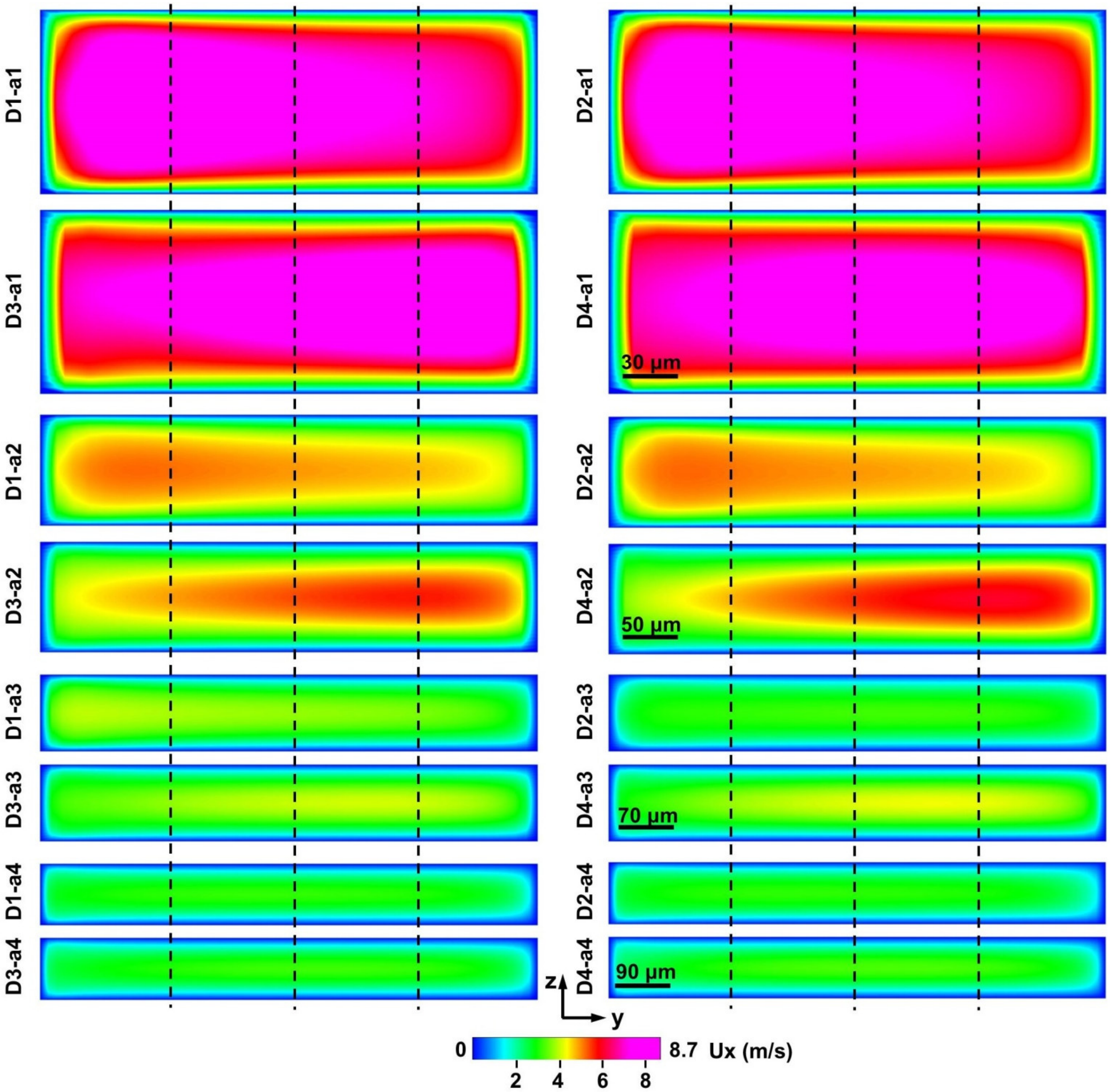

Figure S4. Simulated fluid velocity field in x-axis along channel cross-sections of various narrow regions (NR = 270/900, 450/900, 630/900, 810/900) of D-channels at a flow rate of 15 mL min^{-1}. The a1, a2, a3, and a4 respectively represent 270 μm, 450 μm, 630 μm and 810 μm width of the narrow regions corresponding to the positions in Figure 1A. The cross-sections were quartered for quantitative evaluation of local flow field.

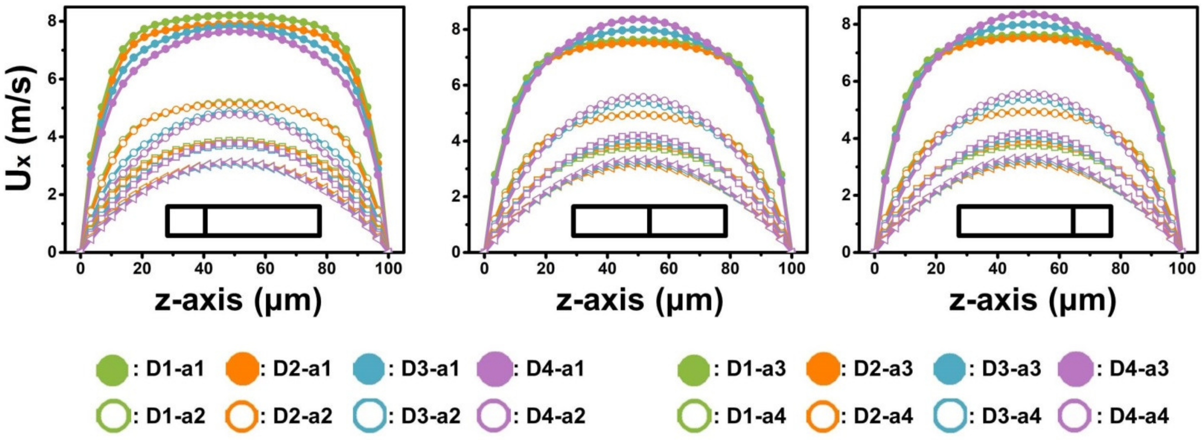

Figure S5. Quantitative analysis of fluid velocity in x-axis along channel cross-sections of various narrow regions (NR = 270/900, 450/900, 630/900, 810/900) of D-channels at a flow rate of 15 mL min^{-1}. The a1, a2, a3, and a4 respectively represent 270 μm, 450 μm, 630 μm and 810 μm width of the narrow regions corresponding to the positions in Figure 1A. The results were acquired from the same position as that marked by the dotted lines (left, middle, right) in Figure S4, respectively.

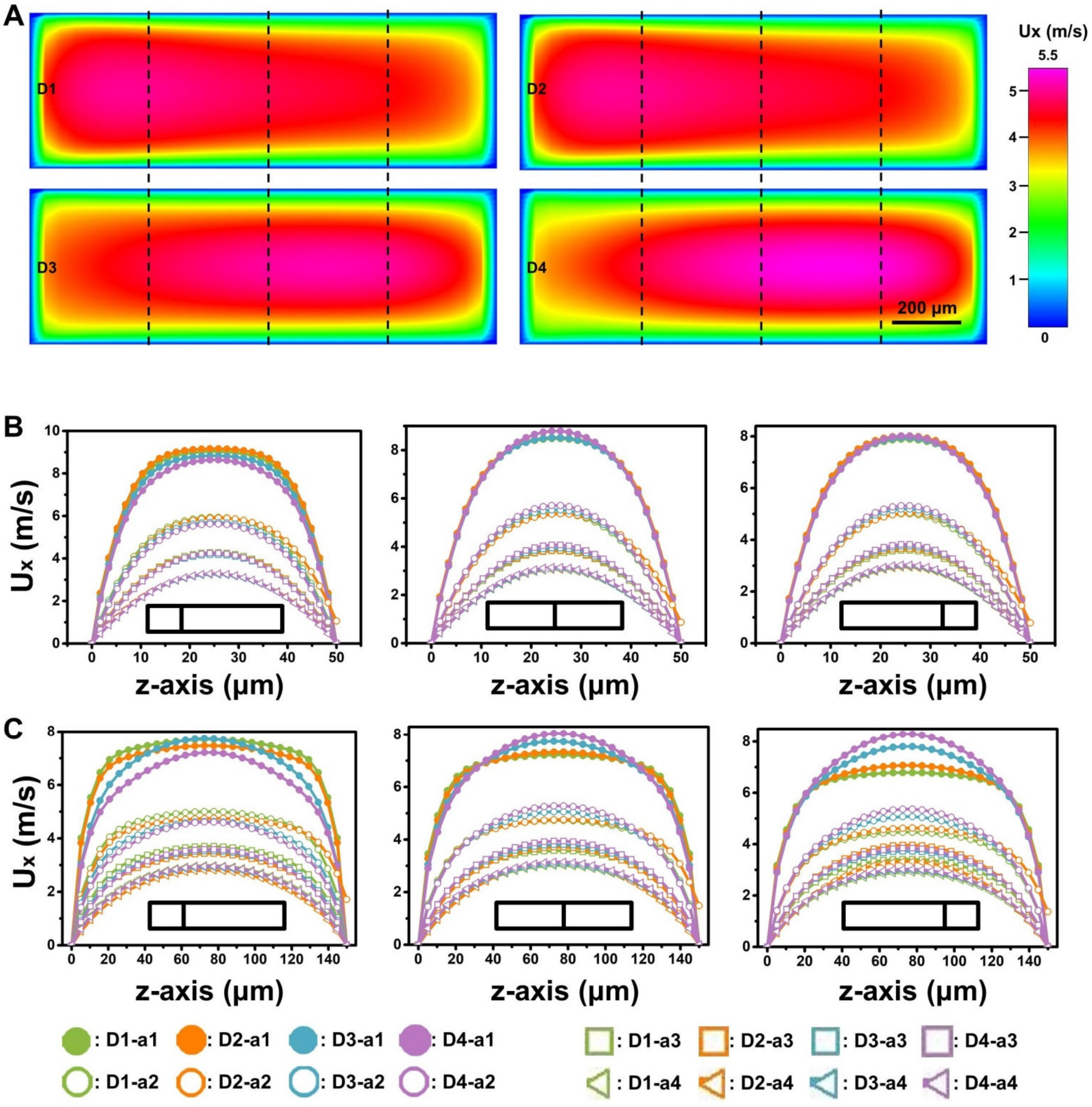

Figure S6. Numerical simulation of fluid motion along channel cross-sections of narrow regions of the D-channels at a flow rate of 15 mL min^{-1}. (**A**) Simulated fluid velocity field in x-axis of the narrow region (NR = 450/900) of different D-channels (D1, D2, D3, D4) with 150 μm height. The cross-sections were quartered for quantitative evaluation of local flow field. (B, C) Quantitative analysis of fluid velocity in x-axis of various narrow regions (NR = 270/900, 450/900, 630/900, 810/900) of different D-channels (D1, D2, D3, D4) with 150 μm (**B**) and 50 μm (**C**) height. The a1, a2, a3, and a4 respectively represent 270 μm, 450 μm, 630 μm and 810 μm width of the narrow regions corresponding to the positions in Figure 1A. The partial results were acquired from the same position as that marked by the dotted lines (left, middle, right) in Figure S6A, respectively.

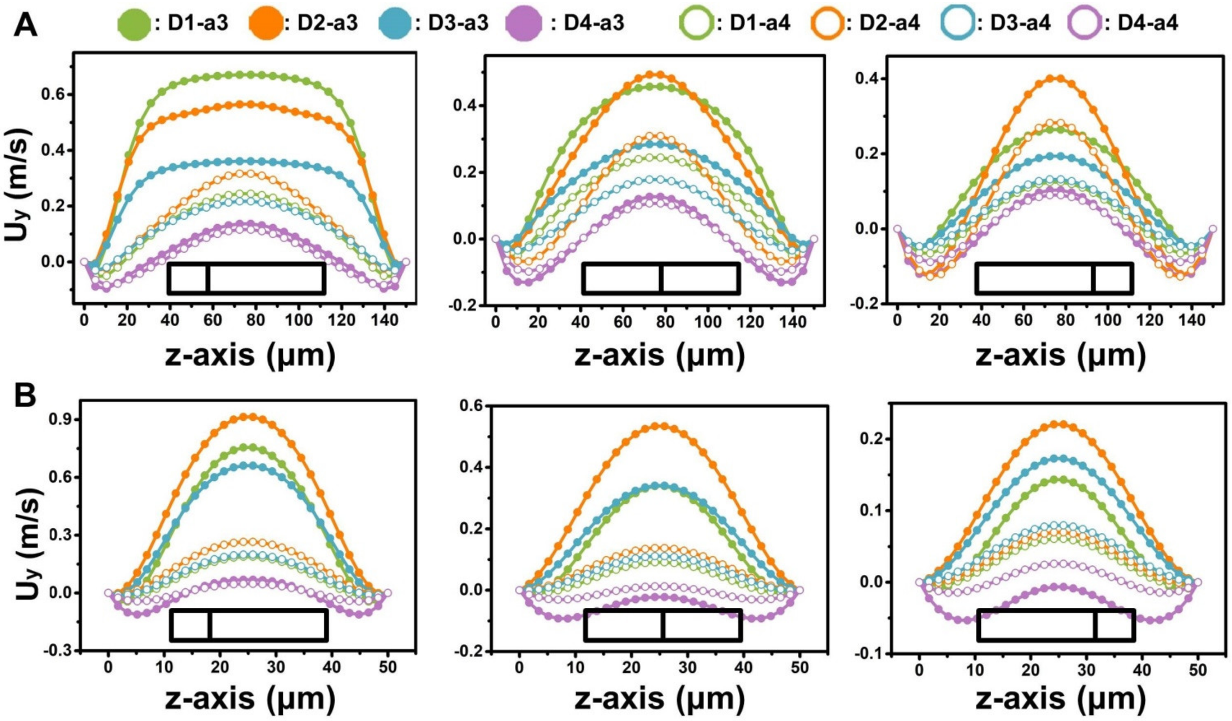

Figure S7. Quantitative analysis of fluid velocity in y-axis of various narrow regions (NR = 270/900, 450/900, 630/900, 810/900) of different D-channels (D1, D2, D3, D4) with 150 μm (**A**) and 50 μm (**B**) height at a flow rate of 15 mL min^{-1}. The a3 and a4 respectively represent 630 μm and 810 μm width of the narrow regions corresponding to the positions in Figure 1A.
